# Addressing Threats to Research and Global Engagement in Human Biology

**DOI:** 10.1002/ajhb.70116

**Published:** 2025-08-22

**Authors:** William R. Leonard, Noël Cameron

**Affiliations:** ^1^ American Journal of Human Biology, Official Journal of the Human Biology Association USA; ^2^ Annals of Human Biology, Official Journal of the Society for the Study of Human Biology UK

**Keywords:** enlightenment, global health, human diversity, international development, research policy, sophisticated

Human Biology is a young science asking the oldest of questions: Who are we? From what did we evolve? How did we deal with threats to our existence? What physiological and morphological adaptations did we develop that allowed our survival and fitness through evolutionary time? In the 21st century, after over three million years of human evolutionary change, what biological characteristics allow us to deal with the existential onslaught that is now affecting our daily lives?

These questions are not just interesting in their own right, but their answers are fundamental to the future of our species and are critically important to addressing long‐standing inequities in health outcomes among human populations around the world. Arguably, the most important characteristic of *Homo sapiens* is our ability to acquire and use knowledge to understand the world around us. In that acquisition, it is the identification of the problem, the sharing of the search, and the answers that allows our onward existence. Threats to the research that targets those specific questions are a threat to humanity.

Since the dawn of the enlightenment in the 17th century, the speed with which we have been able to acquire and use knowledge has gathered pace, requiring ever more sophisticated methods of the sharing and transfer of knowledge through education so that the next generation profits from our endeavors and continues the journey.

We believe that threats exist to the educational and research methods that are the basis of our academic freedom to explore existential questions. These threats to science and promotion of conspiracy theories must be identified and rejected.

The consequences of the actions by the Trump administration are being felt across academia, research institutions, international development agencies, and in the broader global scientific community. As international organizations of Human Biologists, we believe that our collective voice must be heard at this time of crisis to argue for and create a constructive future pathway and not one that destroys the very advantages that have made our species so successful. We believe that government policies that emphasize deregulation, budget cuts, and restrictions on research and global engagement in the health and life sciences are ultimately destructive and must be objected to at every juncture.

We join the voices of our colleagues internationally and through the American National Academies of Sciences, Engineering, and Medicine when we agree that while we hold diverse political beliefs, we are united as researchers in wanting to protect independent scientific inquiry for the future of our species.

Statement of Editorial Independence: The views expressed here are solely those of the authors and do not necessarily represent those of the publisher.

## Data Availability

Data sharing is not applicable to this article as no new data were created or analyzed in this study.

